# The addition of PSMA PET in selecting candidates for prostate cancer focal therapy

**DOI:** 10.1002/bco2.70264

**Published:** 2026-08-06

**Authors:** Kylie Yen‐Yi Lim, Mohammad Asghari‐Jafarabadi, Arveen A. Kalapara, Julian Smith, Mark Frydenberg, Weranja Ranasinghe

**Affiliations:** ^1^ Department of Surgery, School of Clinical Sciences Monash University Clayton Victoria Australia; ^2^ Department of Urology Cabrini Health Malvern Victoria Australia; ^3^ Cabrini Research Cabrini Health Malvern Victoria Australia; ^4^ School of Public Health and Preventive Medicine Monash University Clayton Victoria Australia; ^5^ Department of Psychiatry, School of Clinical Sciences Monash University Clayton Victoria Australia; ^6^ Department of Anatomy and Developmental Biology Monash University Clayton Victoria Australia

**Keywords:** focal therapy, patient selection, prostate cancer, PSMA PET

## Abstract

**Objectives:**

This study aims to evaluate whether prostate‐specific membrane antigen (PSMA) PET improves detection of index lesions and refines patient selection for focal therapy (FT) compared with standard diagnostic pathways.

**Patients and methods:**

We performed a retrospective review of men who underwent radical prostatectomy (RP) between 2015 and 2023. Pre‐operative assessment included multiparametric MRI, PSMA PET/CT and transperineal prostate biopsy. RP whole‐mount histopathology served as the reference standard. Focal therapy eligibility was assessed, and diagnostic performance compared between standard‐of‐care selection criteria and criteria incorporating PSMA PET.

**Results:**

Sixty patients (26%) met SOC criteria for FT. On final RP histopathology, only 20/60 (33.3%) were truly eligible, with discordance most commonly due to bilateral clinically significant prostate cancer (80%). Incorporation of PSMA PET into the selection model improved specificity for appropriate FT selection from 80.3% to 89.7% and positive predictive value from 33.3% to 46.2%. For detection of outfield disease, addition of PSMA PET increased sensitivity to 75.5% and negative predictive value to 53.5%. Higher SUVmax was significantly associated with FT ineligibility (AUC 0.67), with an optimal cut‐point of SUVmax > 4.78 predicting unsuitable candidates.

**Conclusion:**

Only a small proportion of men fulfil criteria for focal therapy, with a high risk of bilateral or contralateral disease using MRI and biopsy alone. PSMA PET improves diagnostic accuracy by identifying outfield lesions and reducing inappropriate FT selection. These findings support integration of PSMA PET to optimise patient selection, with further studies in prospective focal therapy cohorts required.

## 1 INTRODUCTION

Prostate cancer is the most commonly diagnosed cancer in Australia, contributing to a significant cause of cancer‐related death. While radical therapies of prostate cancer are effective, they are associated with significant morbidity, such as urinary incontinence and erectile dysfunction. This has led to a shift in less invasive approaches that maintain oncological control while preserving quality of life. Focal therapy (FT) is an emerging approach that aims to target index lesions to achieve cancer control and reduce treatment‐related side effects. However, accurate identification of tumour location, grade and burden, and therefore patient selection, is critical for FT success.

Although there is a diversity of guidelines available for patient selection, prostate biopsy and MRI currently represent the standard diagnostic tools to guide FT. Nonetheless, these modalities remain imperfect. Despite these pretreatment tests, 21% of patients have cancer recurrence within the treated area, and outfield recurrence occurs in approximately 10%.^1^ While infield recurrence may be suggestive of failure of ablation and outfield recurrence lends to selection failure, ultimately accurate characterisation of tumours is critical. Inadequate characterisation of multifocal, bilateral or clinically significant lesions may lead to compromised treatment outcomes.

Prostate‐specific Membrane Antigen (PSMA) PET imaging has recently transformed the diagnostic and therapeutic landscape of prostate cancer care. Initially introduced as a staging modality for high‐risk disease, PSMA PET has demonstrated superior sensitivity and specificity compared to conventional imaging in detecting both locoregional and metastatic lesions. Beyond staging, there is growing evidence to support its role in primary tumour localisation and characterisation.^2^ It therefore offers to be a valuable adjunct to current diagnostics and refine selection of patients for FT. The aim of this study was to evaluate if PSMA PET can enhance detection rates of index prostate lesions for focal therapy.

## 2 PATIENTS AND METHODS

We conducted a retrospective cohort study examining data from a single surgeon cohort. This study was approved by Monash University Human Research Ethics Committee, Monash Health Human Research Ethics Committee (HREC/97225/MonH‐2023‐368 404) and Cabrini Research Governance (project number Project 01‐03‐08‐23). Data including preoperative MRI, PSMA PET/CT, and transperineal (TP) prostate results were collected of 231 men who underwent a radical prostatectomy (RP) for prostate cancer treatment between April 2015 and April 2023. PSMA PET‐CT was performed prior to TP biopsy or after at the discretion of the treating clinician. RP specimens underwent whole‐mount assessments that involved marking of pathologic lesions.

Standard of care (SOC) eligibility criteria for FT was defined according to a Delphi consensus and applied to assess patient suitability. These included: PSA < 10 ng/mL, International Society of Pathology (ISUP) grade ≤ 3 within the intended treatment area, Gleason 3 + 3 permitted in the untreated region and cancer foci <1.5 mL or <3 mL localised to one hemi‐gland on MRI. RP specimens were defined eligible for FT with a single favourable lesion and ISUP ≤ grade 3. Sensitivity and specificity analyses were performed using RP histopathology as the gold standard. PSMA‐PET positivity was defined as radiologically reported PSMA uptake. Clinically significant prostate cancer was defined as Gleason 7 or more. Discordance was defined only as significant spatial mismatch that would impact treatment planning—specifically, lesions identified in a different region such as the midline or contralateral lobe. Minor adjacent zonal variation was not classified as discordance.

Statistical analysis was conducted using Stata 18 (StataCorp, Texas, USA) using Mean (SD)/Median (Percentile 25–Percentile 75) for numerical variables and using frequency (percent) for categorical variables. Group comparisons were conducted using Fisher's exact tests and Mann–Whitney tests. Diagnostic indices including sensitivity, specificity, positive predictive value (PPV), and negative predictive value (NPV) were calculated.

## 3 RESULTS

Baseline patient characteristics are described in Table 1 with 231 patients who underwent RP for active treatment of prostate cancer. The majority of patients (79.3%) had concerning features of prostate cancer on MRI with a Prostate Imaging Reporting and Data System (PI‐RADS) 4 or 5 lesions reported. One hundred eighty‐seven (81%) patients had ISUP grade group 2 and 3 on transperineal prostate biopsy with a similar percentage reflected in the RP specimens in 197 patients (85.3%).

**TABLE 1:** Baseline patient characteristics.

	*n* = 231
Clinical demographics	
Age, median (IQR) years	68.2 (63.3–72.5)
PSA, median (IQR) ng/mL	6.3 (4.7–8.4)
mpMRI lesions	
Prostate size	34 (25–45)
PIRADS 1/2	23
PIRADS 3	19
PIRADS 4	112
PIRADS 5	76
PSMA‐PET lesions	
Dose, median (IQR) MBq	151 (133–176)
tUptake, median (IQR) minutes	62 (58–68)
SUVMax	5.2
Transperineal prostate biopsy	
Target biopsy	207
ISUP 1	8
ISUP 2	116
ISUP 3	71
ISUP 4	15
ISUP 5	21
Radical prostatectomy	
ISUP 1	1
ISUP 2	115
ISUP 3	82
ISUP 4	3
ISUP 5	30

One hundred ninety‐three patients had a serum PSA ≤ 10 mg/mL. Of these patients, 132 met index cancer volume on MRI, and 131 patients had ISUP ≤ 3 disease on TP biopsy in one index lesion.

Sixty (26%) patients were considered eligible for FT according to the selection criteria. The median age of this cohort was 66.7 years with a median PSA of 5.2 ng/mL (IQR 3.4–6.5) and median prostate volume of 30 cc.

Upon review of RP pathology specimen of these 60 patients, 40 (66.6%) had discordant index tumour characteristics pre‐operatively and on final RP histopathology. Discordance was defined as patients meeting pre‐operative focal therapy (FT) eligibility criteria whose final radical prostatectomy (RP) histopathology demonstrated features rendering them unsuitable for FT. Reasons for FT ineligibility were bilateral clinically significant PCa (csPCa) in 80%, large volume tumours in 12% and extraprostatic extension in 8%.

Logistic regression of continuous variables is summarised in Table 2. There was statistical significance of pretreatment PSA, SUV readings in the right hemi prostate and SUVmax between patients who were eligible and ineligible for FT.

**TABLE 2:** Continuous variable analysis of patients eligible and ineligible for FT and results of logistic regression.

Characteristics	Ineligible for FT median (IQR)	Eligible for FT median (IQR)	OR (95% CI)	*p*val
Age	68 (63.4–72.7)	67 (61.1–70.3)	0.96 (0.91–1.01)	0.134
PSA pretreatment	7 (4.9–8.7)	5 (3.0–6.5)	0.76 (0.64–0.91)	0.003
MRI				
Prostate size	34 (25.0–45.0)	33 (24.0–45.0)	0.99 (0.97–1.01)	0.475
PI‐RADS	4 (4.0–5.0)	4 (4.0–4.0)	0.70 (0.47–1.03)	0.073
Left lesion size (cm)	1 (0.9–1.8)	1 (0.6–1.1)	0.28 (0.08–0.95)	0.042
Right lesion size (cm)	1 (1.0–2.0)	1 (0.8–1.2)	0.26 (0.07–1.00)	0.050
PSMA PET				
SUV left side	4 (2.7–6.4)	4 (2.7–4.8)	0.92 (0.80–1.05)	0.214
SUV right side	4 (3.1–6.3)	3 (2.7–4.3)	0.75 (0.57–0.98)	0.036
SUVmax	5 (3.9–8.9)	4 (3.0–5.2)	0.83 (0.70–0.97)	0.023

**FIGURE 1:**
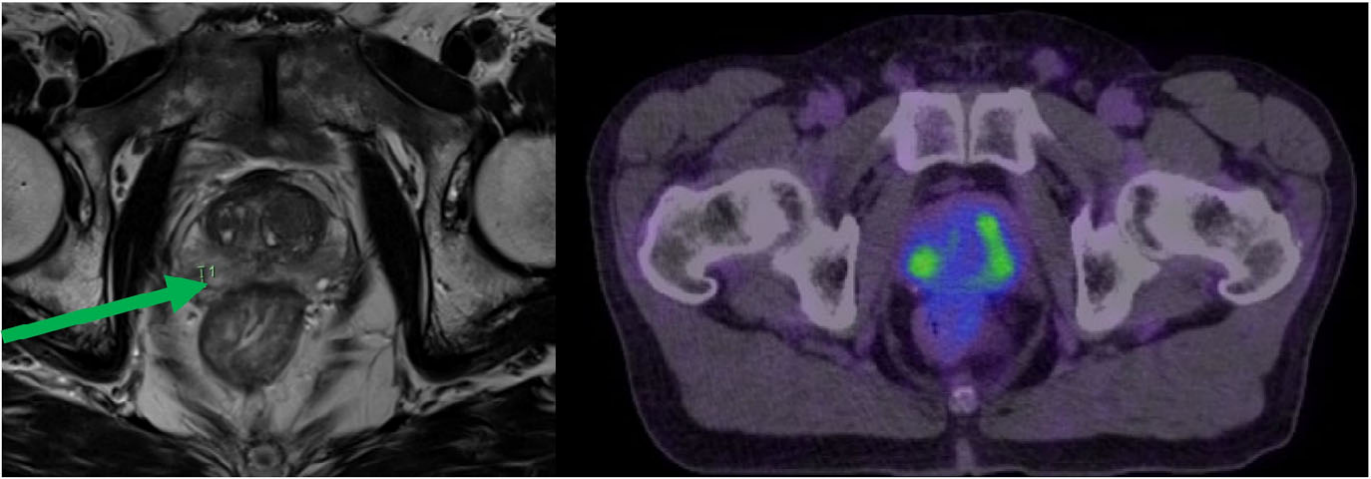
Example of missed outfield lesion, with focal lesion identified on T2 MRI imaging at right posterolateral peripheral zone, compared to multifocal PSMA uptake on PSMA PET.

The addition of PSMA to the FT selection model improved specificity from 80.3% to 89.7% and PPV from 33.3% to 46.2% (Table 3). The addition of PSMA in detecting outfield prostate cancer lesions improved sensitivity to 75.5% and NPV to 53.5% with a decrease of specificity from 91.2% to 67.6% (Figure 1, Table 4). Total accuracy demonstrated that PSMA improved selection and outfield detection.

**TABLE 3:** Diagnostic indices of Delphi selection criteria and Delphi with PSMA in selecting appropriate patients for FT.

	Delphi accuracy	Delphi with PSMA
Sensitivity	71.4%	64.3%
Specificity	80.3%	89.7%
PPV	33.3%	46.2%
NPV	95.4%	94.8%
Accuracy (95%CI)	79.22 (73.41–84.26)	86.58 (81.5–90.7)

**TABLE 4:** Diagnostic indices of Delphi selection and Delphi with PSMA in detecting outfield lesions.

	Delphi out‐of‐field lesions detection accuracy	Delphi with PSMA
Sensitivity	55.8%	75.5%
Specificity	91.2%	67.6%
PPV	93.8%	84.8%
NPV	46.3%	53.5%
Accuracy (95%CI)	66.23 (59.74–72.3)	73.16 (66.96–78.76)

**FIGURE 2:**
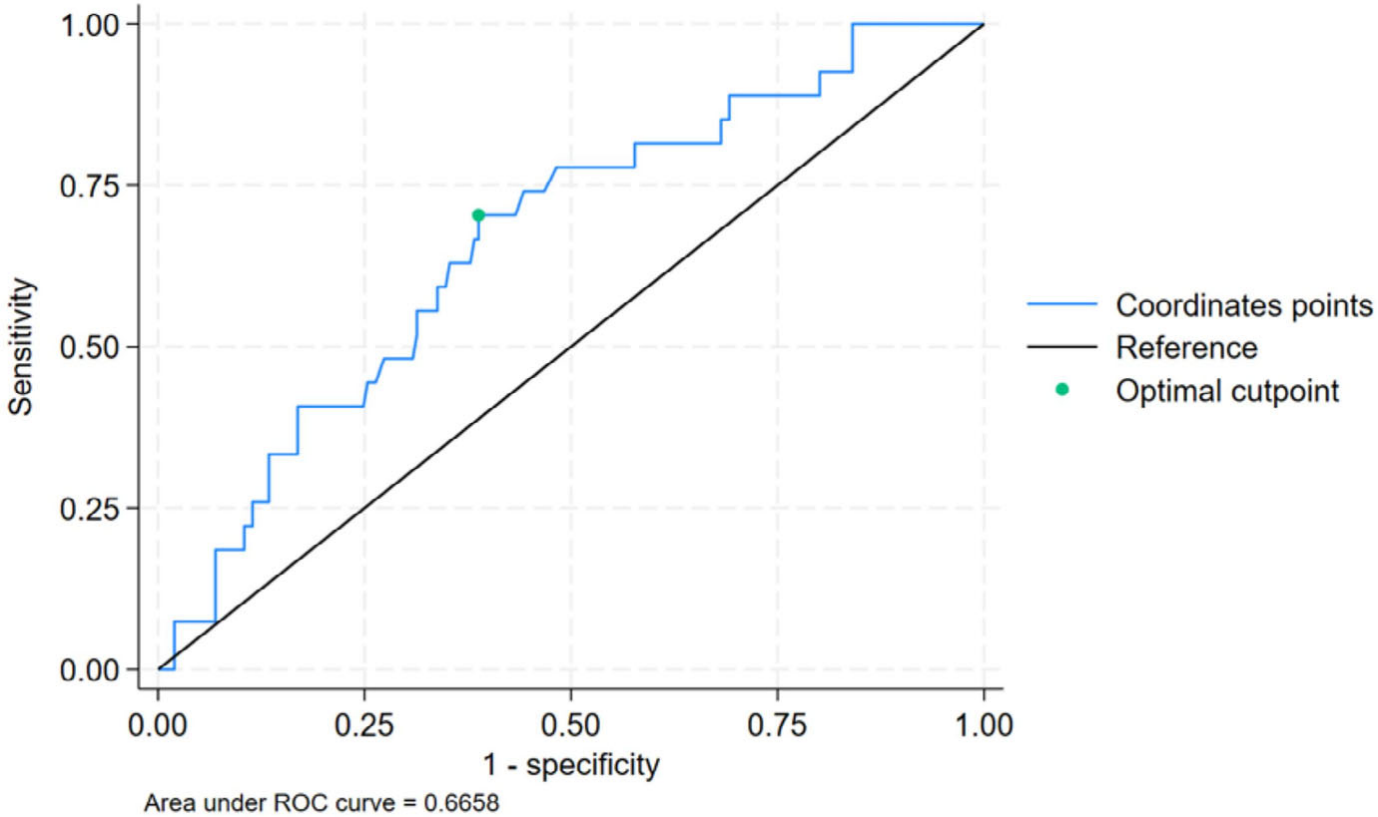
Receiver operating characteristic (ROC) analysis of SUVmax in predicting FT eligibility.

Further analysis of PSMA PET in the accuracy of patient selection demonstrated that SUVmax was negatively correlated with FT eligibility. AUC (95%CI) for SUVax was 66.58 (56.08–77.09) (Figure 2). The optimal cutpoint based on the maximum Youden index for was SUVmax > 4.78 in predicting ineligibility for FT. Binary logistic regression was performed to assess whether SUVmax in the index lesion was associated with upgrading to higher grade cancer at RP compared with biopsy grade. The mean SUVmax was higher among patients with upgrading compared to those without upgrading, although the difference was modest (7.79 [SD 4.81] vs. 7.00 [SD 6.24]). In univariable logistic regression, SUVmax was not significantly associated with upgrading (OR 1.019, 95% CI 0.959–1.072; *p* = 0.487). After adjustment for age, log‐transformed PSA and prostate size, SUVmax remained non‐significant (adjusted OR 1.013, 95% CI 0.952–1.066; *p* = 0.641).

Logistic regression was used to evaluate whether SUVmax of outfield lesions was associated with clinically significant outfield cancer on radical prostatectomy in patients who did not have outfield cancer identified on pre‐RP template biopsies. Mean SUVmax of outfield lesions was higher among patients with csPCa outfield cancer on RP compared with those without csPCa outfield cancer (3.20 [SD 3.00] vs. 2.21 [SD 1.56]). In univariable analysis, SUVmax of outfield lesions was significantly associated with csPCa outfield cancer on RP (OR 1.262, 95% CI 1.040–1.589; *p* = 0.034). After adjustment for age, log‐transformed PSA and prostate size, SUVmax of outfield lesions remained independently associated with csPCa outfield cancer on RP (adjusted OR 1.253, 95% CI 1.032–1.580; *p* = 0.041). This indicates that each one‐unit increase in SUVmax was associated with approximately 25% higher odds of csPCa outfield cancer on RP.

## 4 DISCUSSION

Our analysis demonstrates that a significant proportion of patients were not eligible for FT. In our cohort, only 8.7% met eligibility criteria, consistent with findings from von Hardenberg et al. that have reported 12.8% of patients were potential FT candidates.^3^ Their study applied similar selection parameters but allowed a higher PSA threshold of <15 ng/mL. Applying a stricter PSA cut‐off to <10 ng/mL excluded an additional 2.5% of patients. This highlights the considerable challenge for clinicians to accurately identify and select appropriate candidates from a small cohort and emphasises the need for precise selection criteria and diagnostic tools.

Beyond the limited number of potential candidates, accurate patient selection poses an additional challenge. Among those deemed suitable for FT in our study, only 33.3% were confirmed as truly eligible following pathological review of RP specimens. This level of discordance is similar to a retrospective review of 665 patients who had a RP, which found that 48% of patients initially considered eligible for hemiablation therapy would have in fact been inadequately treated for reasons similar to our study.^4^ These were tumours that were bilateral or crossing the midline and would therefore have left incompletely treated cancer or contralateral csPCa. These results demonstrate that despite multiparametric MRIs and systematic template biopsies, there remains a significant risk of untreated csPCa after FT. While patients undergoing FT are subject to close post‐treatment surveillance and current evidence indicates that focal therapy preserves the effectiveness of subsequent salvage treatments, the presence of untreated outfield csPCa represents a significant burden of residual disease, with inherent risk of progression prior to detection.

A methodological distinction between our study and the above retrospective study is transrectal ultrasound‐guided prostate biopsies were performed to determine hemiablative candidacy, compared to our cohort who had TP prostate biopsies. The Delphi consensus does not distinguish between TRUS or TP biopsies, with MRI‐TRUS fusion biopsies considered acceptable. However, TP biopsy offers several advantages including reduced risk of infections and access to the whole prostate, reflected in the increasing movement towards TP biopsies. In particular, the diagnostic superiority of TP template‐guided biopsies (TTMB), with detection of bilateral disease and upgrading of Gleason staging compared to TRUS has been highlighted by Onik and Barzell.^5^ Furthermore, treatment selection between our cohorts is not entirely comparable, as our cohort utilised PSMA PET in the pretreatment setting that may have influenced the decision to proceed with RP in some patients. In addition, all RP specimens in our study were reviewed by specialised uropathologists, which may have further influenced the accuracy of pathological assessment.

Bilateral csPCa cancer missed was the most common cause of discordance, accounting for 80% of cases in our study. This finding is supported by a retrospective review of whole‐mount RP specimens for localised small‐tumour volume prostate cancer that found that 37% of cases had bilateral disease and multifocal disease in 69%.^6^ However, in this study, it is unclear of the Gleason scoring of the contralateral and multifocal disease. The authors speculate that bilateral foci in small‐volume tumours may be indicative of field cancerisation, which pose a higher risk of other tumours arising if left untreated.

Our results show that adding PSMA PET imaging improved the specificity of patient selection and improved identification outfield lesions, thereby preventing inappropriate FT. In particular, higher SUVmax in outfield lesions was independently associated with clinically significant outfield cancer on RP histopathology. These findings further support a potential role for PSMA PET not only in improving FT candidate selection, but also in identifying biologically significant outfield disease that may otherwise lead to undertreatment. A systematic review on the role of PSMA PET in pre‐operative planning for FT was recently published, highlighting a scarcity of studies.^7^ One of the five studies included was a prospective study of 17 patients that examined the utility of 18F‐Florastamin PSMA‐PET/CT prior to IRE prostate cancer treatment. It was reported that PSMA PET revealed an ISUP positive lesion that was missed on mpMRI, supporting our results that PSMA adds value in detecting missed lesions.^8^

Further support of the addition of PSMA PET comes from a retrospective analysis of 138 patients with ISUP grade 2–3 prostate cancer who underwent a RP.^9^ The diagnostic accuracy of mpMRI, systematic biopsies and PSMA‐PET in correctly identifying patients suitable for hemi‐ablative FT were analysed. Their results confirmed that PSMA‐PET improved accuracy for csPCa with increased sensitivity to 87% and negative predictive value of 86%. In this study, PSMA PET/CT was performed either prior or post‐prostate biopsy. However, this study by Geboers et al demonstrated that although PSMA‐PET was performed post‐biopsy, there was a low false positive rate of 3% which assures that the accuracy of PSMA‐PET is not affected by post‐biopsy tissue inflammation.

The diagnostic benefit of PSMA PET has been confirmed in the multicentre, prospective PRIMARY trial, which demonstrated that combining PSMA PET with MRI significantly increased sensitivity and negative predictive value for csPCa compared with MRI alone.^10^ Collectively, these findings suggest a shift in prostate cancer diagnostics, with MRI and PSMA PET increasingly recognised as complementary tools and likely to become standard‐of‐care workup for FT eligibility.

This study has several limitations. It is retrospective and based on a single‐surgeon cohort, which may limit generalisability. The cohort was heterogenous with a spectrum of prostate cancer grades. Furthermore, there was selection bias given that the patient cohort had surgical treatment and there may be patients who underwent active surveillance or radiation therapy who may also be considered eligible for focal therapy, who were not captured. Nonetheless, this cohort was selected as RP specimens allowed true gold standard comparison. As this analysis was theoretical, further prospective studies of patients receiving FT may provide further evidence to support the value of PSMA PET. Furthermore, although PSMA PET adds clear diagnostic value, limitations should also be acknowledged. Access remains limited outside tertiary centres, and cost‐effectiveness analyses specific to FT selection are lacking. False negatives may still occur, particularly in low‐PSMA‐expressing tumours, while indeterminate uptake in benign conditions can generate false positives.^11^ These caveats reinforce that PSMA PET should complement, rather than replace, MRI and biopsy in FT candidate selection.

## 5 CONCLUSIONS

Only a small proportion of patients are truly eligible for focal therapy, with significant risk of untreated bilateral or contralateral disease despite MRI and systematic biopsies. Our results demonstrate that PSMA PET improves diagnostic accuracy by detecting outfield lesions and reducing inappropriate selection, consistent with emerging evidence. While retrospective and limited to a surgically treated cohort, this study supports the integration of PSMA PET alongside MRI and TP biopsies to refine selection criteria. Prospective studies in focal therapy cohorts are required to confirm its role in routine practice.

## AUTHOR CONTRIBUTIONS

Kylie Yen‐Yi Lim: conceptualisation; data curation; writing—original draft preparation; writing – review and editing. Mohammad Asghari‐Jafarabadi: Formal analysis. Arveen A. Kalapara: Data curation. Julian Smith, Mark Frydenberg, Weranja Ranasinghe: Writing—review and editing. All authors have read and agreed to the published version of the manuscript.

## CONFLICT OF INTEREST STATEMENT

The authors declare no conflicts of interest.

## Data Availability

Data sharing is not applicable to this article as no datasets were generated or analysed during the current study.
